# Randomized, double-blind, placebo-controled clinical trial of sublingual immunotherapy in natural rubber latex allergic patients

**DOI:** 10.1186/1745-6215-12-191

**Published:** 2011-08-09

**Authors:** Gabriel Gastaminza, Jaime Algorta, Olga Uriel, Maria T Audicana, Eduardo Fernandez, Maria L Sanz, Daniel Muñoz

**Affiliations:** 1 Department of Allergology, Hospital Santiago-Apostol, Vitoria-Gasteiz, Spain; 2 Department of Biochemistry and Molecular Biology. University of the Basque Country, Leioa, Vizcaya, Spain; 3 Department of Allergology and Clinical Immunology, Clinica Universidad de Navarra, Pamplona, Spain

## Abstract

**Background:**

Natural rubber latex allergy is a common and unsolved health problem. Since the avoidance of exposure is very difficult, immunotherapy is strongly recommended, but before its use in patients, it is essential to prove the efficacy and safety of extracts.

The aim of the present randomised, double-blind, placebo-controlled clinical trial was to assess the efficacy and tolerability of latex sublingual immunotherapy in adult patients undergoing permanent latex avoidance.

**Methods:**

Twenty-eight adult latex-allergic patients (5 males and 23 females), with mean age of 39 years (range 24-57) were randomized to receive a commercial latex-sublingual immunotherapy or placebo during one year, followed by another year of open, active therapy. The following outcomes were measured at baseline and at the end of first and second year of follow-up: skin prick test, gloves-use score, conjunctival challenge test, total and specific IgE, basophil activation test, and adverse reactions monitoring.

**Results:**

No significant difference in any of the efficacy *in vivo *variables was observed between active and placebo groups at the end of the placebo-controlled phase, nor when each group was compared with their baseline values at the end of the two year-study. An improvement in the average percentage of basophils activated was observed. During the induction phase, 4 reactions in the active group and 5 in the placebo group were recorded. During the maintenance phase, two patients dropped out due to pruritus and to acute dermatitis respectively.

**Conclusion:**

Further studies are needed to evaluate latex-sublingual immunotherapy, since efficacy could not be demonstrated in adult patients with avoidance of the allergen.

**Trial registration number:**

ACTRN12611000543987

## Background

Natural rubber latex (NRL) is an ubiquitous material and NRL-allergy is a relatively recently considered diagnosis as it was first recognised in the late 70s [1,2]. It mostly affects certain groups of high risk populations (health care workers, patients with frequent hospitalization) but the prevalence of latex sensitisation in general population is also far from negligible. Moreover, there has been a recent increasing number of cases of NRL-allergy in other occupations including hairdressers, housekeepers, construction workers, food handlers or security personnel [3,4], probably due to widespread exposure to latex gloves. Therefore, NRL-allergy represented a recognised public health concern until corrective measures were adopted (mainly, the substitution of NRL by other materials or the use of powder-free gloves in healthcare institutions), resulting in a progressive decline in the incidence of the disease [5].

The avoidance of NRL is currently recommended to reduce the risk of allergic symptoms (including life-threatening reactions) in patients with established allergy. However, in the case of NRL this measure is clearly insufficient because it is quite difficult to keep away from the widespread presence of latex in many products. It is also complicated due to the cross-reactions of latex with various common fruits like avocado, banana or kiwi [6,7], that is mainly due to Hevein (Hev b 6) sensitisation. Moreover, while long-term avoidance of natural rubber latex can resolve symptoms, detectable IgE indicating continued sensitization remains beyond 5 years [8].

Immunotherapy using a NRL extract could hypothetically protect these patients from an adverse episode after accidental contact to NRL. However, although it is a common sensitization, the availability of different commercial products with proven efficacy and safety is still limited. Only a few studies with the appropriate design (randomized, double-blind and placebo-controlled) have been published regarding the efficacy and tolerance of specific immunotherapy. In fact, there is evidence that subcutaneous immunotherapy leads to a significant improvement in various efficacy parameters when compared to placebo, but with the drawback of a high rate of systemic reactions [9,10].

Sublingual administration of NRL-immunotherapy has also been barely explored in adult patients. Patriarca et al. [11] showed an improvement in skin and respiratory symptoms, and also in the conjunctival challenge test, but this was a non-controlled study. In another open-label and non-controlled study, Cistero [12] demonstrated a better tolerability of a rush sublingual immunotherapy than with the subcutaneous route, but only assessed the efficacy measuring a cutaneous response after 10 weeks. A double-blind, placebo-controlled study of one-year sublingual immunotherapy was published by Nettis [13], showing a significant improvement in symptoms and medication scores after 12 months of treatment. Finally, a very recent trial suggest that latex SLIT was more effective than placebo after one-year follow-up, but only 9 patient completed the study and hence, results must be taken with prudence [14].

Bearing in mind that the sublingual application is better tolerated than the subcutaneous route, more studies on the long-term effect of this therapy are needed, especially when such therapy has been available for years. For this reason, we conducted this independent (sponsored by the investigators) randomised, double-blind, placebo-controlled clinical trial to assess the efficacy and tolerability after 2 years follow-up of a commercially available NRL-sublingual immunotherapy in adult patients undergoing permanent NRL avoidance.

## Methods

### Design and ethics

This is an independent study which was sponsored by the investigators and partially funded by public institutions.

The study was designed in two phases: First, a randomized, double-blind, placebo controlled clinical trial was carried-out. At the end of the first year, follow-up (T1), blinding was discontinued and active-treated patients continued receiving treatment for another additional year (T2). Those patients who were included in the placebo group received active therapy for one year in an open-label manner.

The study was approved by the local Ethic's Committee/Independent Review Board as well as by the Spanish Medicines Agency. The trial was conducted according to Good Clinical Practice, and therefore, prior to the enrolment all subjects gave their written informed consent on the basis of verbal and written information and external monitoring and audit was performed by independent investigators. All study procedures are summarised in Table 1.

**Table 1:** Study procedures

	Screening	Double blind				Open label		
**Inclusion/exclusion criteria**	X							

**Informed consent**	X							

**Active treatment**		X	X	X	X	X	X	X

**Placebo**		X	X	X	X			

**Skin Prick Tests**	X	X	X	X	X	X	X	X

**Glove Use Test**		X			X			X

**Conjunctival Challenge**		X			X			X

**IgE**		X			X			X

**Basophil Activation Test**		X			X			X

**Adverse events**		X	X	X	X	X	X	X

	**Months**	**0**	**1**	**6**	**12**	**13**	**18**	**24**

### Patients

Participation in the study was proposed to the 76 patients allergic to NRL registered in the research centre (Santiago Apostol Hospital, Vitoria-Gasteiz, Spain). Finally, 28 patients (5 males and 23 females), with mean age of 39 years (range 24-57 years-old) accepted to participate and were included. All the patients were previously diagnosed as having latex allergy and were subsequently instructed to avoid exposure to the allergen. The clinical characteristics of the patients are summarized in Table 2.

**Table 2:** Clinical and molecular features of patients recruited

	Active group	Placebo group
Nr. PATIENTS		
Total	14	14
PROFFESION		
Health care	9	8
Other	5	6
SYMPTOMS		
Urticaria	10	11
Rhinoconjunctivitis	10	9
Asthma	7	1
Anaphylaxis	4	2
OTHER SENSITIZATIONS		
Food allergy	7	5
Allergy to aeroallergens	4	6
Mites	4	4
Pollens	2	6
Moulds	2	0
Epithelia	0	2
SPECIFIC IgE		
Median IgE-k82	7.5 kU/L	1.8 kU/L
Median IgE Hev b 5	5.6 kU/L	0.0 kU/L
Median IgE Hev b 6	1.5 kU/L	0.4 kU/L

Inclusion criteria comprised a clinical history of natural rubber latex allergy (documenting the usual symptoms of urticaria, angioedema, rhinitis, conjunctivitis, asthma or anaphylaxis) or a positive response to the gloves use test and/or conjunctival test plus positive prick test to NRL (wheal ≥ 3 × 3 mm). Exclusion criteria consisted of the usual contraindications for the use of immunotherapy (severe or uncontrolled asthma, other immune-mediated diseases, coronary artery disease, or the concurrent use of beta-blockers or angiotensin-converting enzyme inhibitors) [15] and the presence of severe systemic or psychiatric diseases, chronic urticaria or dermographism. Patients meeting all the eligibility criteria were randomly allocated to the active immunotherapy group or placebo group.

### Study treatments

A commercially available sublingual immunotherapy (SLIT-Latex^®^, ALK-Abello, Spain) was assayed in the trial. The medication for the trial was supplied by the manufacturer directly to the Department of Pharmacy at the research centre. It was part of a commercial batch, but labelled for the trial as specified in the Annex XIII of Good Manufacturing Practice Guideline. SLIT-Latex^® ^is a galenic formulation containing latex allergenic extract (*Hevea brasiliensis*), human albumin (except in vial 4), sodium chloride, phenol, glicerine and water. The extract is standardised in accordance with the protein content (SDS-PAGE) and with total protein content. The higher concentration is 500 mcg protein/mL. In addition, placebo was prepared by the same manufacturers and contained the same composition except the allergen extract. Both treatments had the same external appearance, taste and colour. Immunotherapy was administered according to manufacturer's schedule (Table 3). Patients were carefully instructed to held the drops under the tongue for 3 minutes before being swallowed. For safety reasons, according to EAACI, induction phase was administered at hospital setting under the surveillance of a trained allergologist and patient remained under observation for at least 30 minutes after each dose. Successive maintenance doses were self-administered at home. Patients attended the hospital every three months to collect new doses and to return the empty flasks, which were counted by investigators for the assessment of compliance.

**Table 3:** Treatment schedule of Sublingual Latex Immunotherapy: Concentration of vials and build-up dosing

Day	Flask	Concentration (μg/ml)	**Nr**. drops	Dose administered (μg)	Cumulative dose (μg)	Total/day (μg)
**1**	(0)	5.10^-8^	1 10	2.10^-9^ 2.10^-8^	2.10^-9^ 2.10^-8^	
	(1)	5.10^-5^	1 10	2.10^-6^ 2.10^-5^	2.10^-6^ 2.10^-5^	2.10^-5^
**2**	(2)	5.10^-2^	1 10	0.002 0.02	0.002 0.022	
	(3)	5	1 10	0.2 2	0.222 2.222	2.2
**3**	(4)	500	1 2 3 4 10	20 40 60 80 200	22.2 62.2 122.2 202.2 402.2	400
**4**	(4)	500	25	500	902.2	500
		Maintenance				
**5**	(4)	500	2/24 h.	40		40

### *In vivo *tests

**Skin prick tests **to 4 latex concentrations (4, 20, 100 and 500 μg) were performed in duplicate in each study visit. Positive (histamine hydrochloride 10 mg/ml) and negative (saline solution) controls were also included in each test. The areas of the papullae were transferred onto paper and subsequently measured by planimetry using specific software [16]. The mean of the areas of the papullae were calculated for both groups, active and placebo. For comparing the results of prick test, before and after immunotherapy, the cutaneous tolerance index (CTI) was calculated. This index is the factor by which it is necessary to multiply the dose of the extract to obtain a similar cutaneous response and estimates the greater or smaller cutaneous sensitivity of one group in relation to the other group. When it is used to evaluate whether changes are observed in a group of patients, a CTI higher than 1 indicates a reduction in the cutaneous response.

**Glove Use Test (GUT) **was carried out with high content latex gloves (Non-sterile Aachen^®^, Spain) and with vinyl 100% latex-free gloves (Torval^®^, China). Patients were protected with glasses and a latex-free mask in order to avoid concurrent eye and/or inhalative exposure. Patients wore one type of glove (latex or vinyl) on each hand for 5, 15, and 30 minutes, separated by an interval of 20 minutes. Symptoms (pruritus, erythema, wheals) were scored (0 = absent; 1 = mild; 2 = moderate; 3 = severe) as previously described [17]. The test was stopped when a symptom score of 5 was reached. Results are expressed as the estimated time to achieve five points.

**Conjunctival Challenge Test (CCT) **was performed applying five increasing concentrations of a NRL extract (0.08 - 0.4 - 2 - 10 and 50 mg/ml), separated by 15 minute intervals. The test was carried out by placing a drop of the lower dose on the inferior conjunctival fornix of the right eye and one control saline solution drop on the left eye. The test was considered negative if no reaction was seen after 15 minutes, and then the next concentration was added to the right eye. Symptoms of hyperaemia, chemosis, epiphora, pruritus, sneezes, and nasal congestion were scored as previously described [18] (0 = absent; 1 = mild; 2 = moderate; 3 = severe). The test was stopped when a symptom score of 5 was reached. Results are expressed as the estimated concentration to achieve five points.

### In vitro tests

**Specific IgE **to natural rubber latex (k82) and to recombinant allergens (rHev b 1, rHev b 3, rHev b 5, rHev b 6,01; rHev b 8) were measured by a standard system based on fluorescent solid phase enzyme immunoassay (CAP-FEIA^®^, Phadia, Sweden).

**Basophil Activation Test **(BAT): The percentage of basophils that expresses CD63 as an activation marker after *in vitro *stimulation with an allergen extract of latex was determined by flow cytometry, following double labelling with the monoclonal antibodies anti-CD63-PE and anti-IgE FITC. Aqueous sterile filtered standardised extracts of latex devoid of any preservatives at final concentrations of 0.125 and 0.03125 mg protein/ml (BIAL-Aristegui, Bilbao, Spain) were used.

For comparison between BAT results at different times, the mean of the percentage of basophils that expressed CD63 after *in vitro *stimulation with an allergen extract of latex at final concentrations of 0.125 and 0.03125 mg protein/ml were calculated.

### Safety

Since the induction phase was carried out in the hospital and the patients remained under direct medical surveillance during 30 minutes after treatment administration, immediate adverse events were directly recorded in the Case Report Form. In addition, heart rate, arterial pressure and peak-flow measurement were measured before and after the administration of a dose. During the maintenance phase, patients were instructed to annotate any inconvenience on a diary card. Safety was monitored by the investigators each study visit, and in addition, the patient always had the opportunity to contact the allergologist or to attend the emergency room in case of severe adverse reactions. Adverse reactions were classified according to European Academy of Allergy, Asthma and Clinical Immunology (EAACI) recommendations [14].

### Statistical methods

Changes in cutaneous reactivity by skin prick test were analysed using a parallel lines assay [19] and the ALASA CRS PLA software (Madrid, Spain) [16]. The differences were expressed using the Cutaneous Tolerance Index (CTI), calculated as above was described. The CTI is expressed with the 95% and 99% confidence intervals. When CTI is applied across groups, it estimates the greater or smaller cutaneous sensitivity of one group in relation to the other group. When it is used to evaluate whether changes are observed in a group of patients, a CTI higher than 1 indicates a reduction in the cutaneous response.

For the evaluation of Glove Use Test, a linear regression line symptom's score/time was calculated in each patient to individually estimate the time to reach five points. In a similar procedure, for the evaluation of Conjunctival Challenge Test, the individual linear regression symptom's score/concentration was calculated to individually estimate the concentration necessary to reach five points.

Comparisons between groups and within groups were performed through the non-parametric Mann-Whitney's and Wilcoxon's tests, respectively. Significance was set at p < 0.05.

## Results

**Figure 1:**
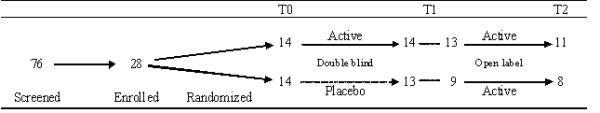
**Allocation of patients to treatment group and study phases**.

Twenty seven out of the 28 patients initially enrolled completed the first year of follow-up. The remaining subject dropped out due to personal reasons not related with the trial. At the end of the double-blind trial, one patient in the active group and 4 patients in the placebo group declined to participate in the subsequent open-label phase with active treatment. During the second year, another three patients dropped out and finally 19 patients completed 2 years of follow-up (Figure 1). In summary, 11 patients received active immunotherapy for 2 years and 22 patients received active immunotherapy for one year (14 during the first year and 8 during the second year).

Before treatment was begun, cutaneous sensitivity to NRL in the active group was similar to that in the placebo group (CTI 2.45; 95%CI: 0.59-13.95). After the one-year double-blind period, there was no significant variation in cutaneous sensitivity to the allergen with the active immunotherapy (3.11; 95%CI: 0.87-15.26).

**Figure 2:**
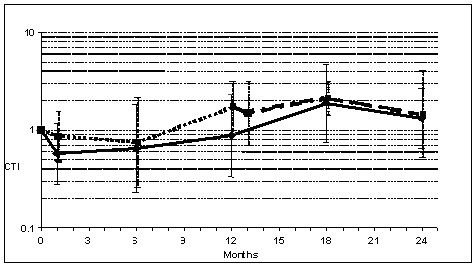
**Results of Cutaneous Tolerance Index (CTI) in Active and Placebo groups during the two years of study**.

When intra-group CTIs (Figure 2) were evaluated, comparing T1 and T2 results with the initial value (T0), in the active group no difference was seen after one year (0.88; 95%CI: 0.33-2.37) or 2 years active immunotherapy (1.32; 95%CI: 0.66-2.63). In the placebo group, no differences were observed after the first year of placebo therapy (1.71; 95%CI: 0.92-3.18) nor after the 2 years of follow-up, the last under active immunotherapy (1.45; 95%CI: 0.52-4.05). When only the year of active immunotherapy was evaluated in the placebo group (T2/T1 comparison), also no differences were found (0.65; 95%CI: 0.18-2.35).

At time of diagnosis, all but one patient were positive to at least one of the challenge tests. This patient fulfilled inclusion criteria because had suffered from symptoms after contact with latex in the last two years. GUT was positive in 24/28 and CCT was positive in 26/28 patients. During the follow-up, no improvement in either GUT or CCT response could be observed, and the number of patients improving was similar to the number of patients worsening in both the Active or Placebo group (Table 4). There were no differences in the mean of the NRL-concentration in CCT to obtain 5 points of symptoms (CCT-5) between active (16.6 mg/ml; 95%CI 5.8-27.3) and placebo (24.4 mg/ml; 95%CI 4.2- 44.5) groups in T0. A non significant improvement was observed comparing the mean CCT-5 in T0 and T1 both in Active (mean CCT-5 at T1: 27.1 mg/ml; 95% CI 5.8-48.3) and in Placebo groups (mean CCT-5 at T1: 35.2 mg/ml; 95% CI 0-71.9). No statistical differences were observed between Active and Placebo at T1.

**Table 4:** Evolution of Glove Use Test (GUT) and Conjunctival Challenge Test (CCT) tests during follow-up period

	GUT		CCT	
	**Active**	**Placebo**	**Active**	**Placebo**

T0				
Nr. Positives/Total	13/14	11/14	14/14	12/14
T1				
Nr.Improving/Total	6/14	4/12	7/14	6/13
Nr.Worsening/Total	2/14	5/12	2/14	2/13
Nr. No changes/Total	6/14	3/12	5/14	5/13
T2				
Nr.Improving/Total	3/11	2/9#	7/11	5/8#
Nr.Worsening/Total	6/11	4/9#	1/11	0/8#
Nr. No changes/Total	2/11	3/9#	3/11	3/8#

# Comparing T2 with T1, after 1 year of active therapy.

There were no differences in the mean of the time in minutes in contact to the glove in GUT to obtain 5 points of symptoms (GUT-5) between Active (23.8 minutes; 95%CI: 1 - 52) and placebo (28.6 minutes; 95%CI: 4.3 - 52.8) groups in T0. An improvement was observed comparing the mean GUT-5 in T0 and T1 only in Placebo group (mean GUT-5 at T1: 64.0 minutes; 95% CI: 0.1 - 130.4); in Active group the mean of GUT-5 at T1 (18.5 minutes; 95% CI: 8.7 - 28.3) got worsen. No statistical differences were observed between Active and Placebo at T1.

Among the 28 enrolled subjects, at time of diagnosis 27 presented a positive level to IgE-k82, 15 to IgE-Hev b 5 and 19 patients were positive to IgE-Hev b 6. Only 2 patients were positive to the other recombinant allergen tested (IgE-Hev b 1, IgE-Hev b 3, IgE-Hev b 8).

**Figure 3:**
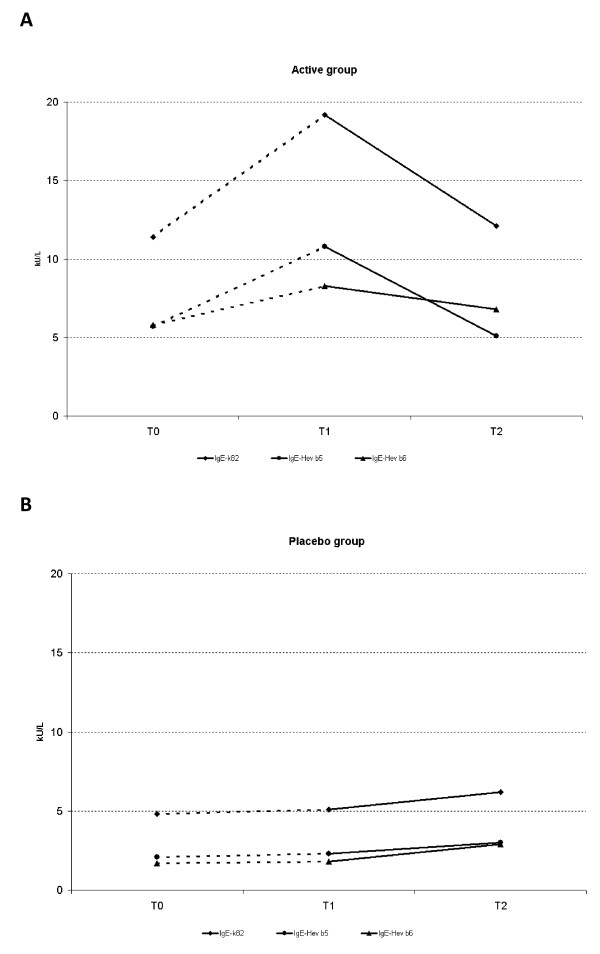
**A. Mean IgE-k82 (black square), IgE-rHev b 5 (black circle), and IgE rHev b 6 (black triangle), during the 2-years follow-up, in active group; B. Mean IgE-k82 (black square), IgE-rHev b 5 (black circle), and IgE rHev b 6 (black triangle), during the 2-years follow-up, in placebo group**.

No changes were observed in specific IgE levels (k82, Hev b 5, Hev b 6) comparing active with placebo groups at any investigational time (T0, T1 or T2). A slight but not significant increase followed by a decline to baseline values throughout treatment was observed in the Active group (Figure 3). In the Placebo group, no changes were seen during the first year, but a non-significant increase was observed after the second year with active therapy, similar to the other group.

At the beginning of the study, BAT was positive in 14/14 patients in the Active group and in 12/14 in the Placebo group. No changes were observed at the end of the double-blind trial, when 12/14 and 12/13 patients, respectively, proved positive. However, at the end of follow up, a clear improvement was demonstrated in both Active group (6/11 positive subjects; 4 negative and 1 not evaluable) and in Placebo group (after only one year of active immunotherapy, 4/8 positive patients, 3/8 negative and 1 not evaluable). No statistical differences were observed comparing the mean of percentage of basophils activated with the NRL concentration of 0.03 mg/mL between active and placebo groups at T1 (data not shown).

The average percentage of basophils activated decreased in the Active group after the 2 years of treatment, and also in the Placebo group only after the second year of active treatment. Also, the percentage of patients in whom BAT fell was higher according to the number of years of active treatment (Table 5).

**Table 5:** Results of Basophil Activation Test (BAT) in all the patients

	N. of years in active treatment		
	**0**	**1**	**2**

fall	2	11	8

raise	11	10	2

total	13	21	10

**% that fall**	**15%**	**52%**	**80%**

Number and percentage of the patients whose BAT results fell or rose after T0 (patients from the Placebo group at T1), 1 year (the sum of the patients from the Placebo group at T2 and Active group at T1), or 2 years of active treatment (Active group at T2).

No severe adverse events occurred and all the patients reached the maximum dose. A total of 9 mild adverse events were recorded during the induction phase, 5 in four patients from the Placebo group during the buid-up of the placebo treatment (1 conjunctivitis, 1 rhinorrhoea, 1 pruritus, 1 dyspnoea, 1 tongue pruritus) occurred in the first doses of the immunotherapy; and 4 reactions in 4 patients from the Active group, that appeared when high doses of an allergen extract of latex were reached (1 tongue pruritus, 3 rhinoconjunctivitis). The build-up schedule was modified only in one patient corresponding to the Placebo group.

During the maintenance phase, one patient was withdrawn after the first year of active treatment due to cutaneous pruritus over the previous months. Another patient who switched from placebo to active treatment in the second year of the study, suffered from an outbreak of acute dermatitis in the feet after one week of maintenance, and was then dropped from the study.

## Discussion

The increase in latex allergy incidence observed in the 80s was associated with the 25-fold increase in the use of latex gloves in order to prevent the transmission of increasing incidence of transmission viral diseases (mainly HIV, BHV and CHV) [20]. Although natural rubber latex exposure dramatically diminished in the sanitary environment, thanks to its functional properties, latex is the basis for manufacturing a large variety of products and there is even now a widespread distribution of latex-containing products in other professional (hairdressers, food-service workers, cleaning workers, police officers) or personnel settings, including sports [21]. Therefore, due to the difficulties to completely avoid the allergen, it is recommended the specific desensitization of those patients, mainly with potential occupational exposure, because it would be expected that in the case of accidental exposure, the reaction would be less severe than in non-hyposensitized allergic patients.

Sublingual administration can be an advantageous alternative to the classical subcutaneous immunotherapy, its efficacy and safety should be distinctively demonstrated for each allergen. In this study, a commercially available sublingual immunotherapy with scarce evidence of efficacy and safety was evaluated through an appropriate randomized and blinded design. It must be highlighted that this was an independent clinical trial, founded only by public funds. Nevertheless, the study was carried out according with Good Clinical Practice guidelines, including appropriate monitorization by external investigators.

Randomized, controlled and blind clinical trials are the standard for the evaluation of new medicines. However, since the sublingual therapy was commercially available, a placebo-controlled trial over a 2 years period was considered not ethically appropriate. In consequence, a combined design was chosen, comprising a first year of double-blind placebo-controlled treatment followed by an additional year as open-label follow-up. This design allowed the assessment of both, the comparison of active and control groups during the first year as well as evaluation of the evolution of the active therapy during 2 years.

A common difficulty in clinical research is the enrollment of patients. In our study, all the latex allergic patients diagnosed at the research centre were directly contacted to requested their participation in the trial. The number of patients finally evaluated can be considered to be truly representative and it is noteworthy that more than 1/3 of the total population of NRL allergic patients registered in the Health Area finally participated in the study. However, the number of withdrawals in the switch from the double-blind to the open-label phase was higher than expected. This can be partly attributed to a subjective perception of the lack of efficacy and partly to the low frequency of symptoms and subjective feeling of not needing treatment. In spite of two patients who abandoned the study because they suffered an acute recurrence of skin symptoms (both had a history of former atopic dermatitis). The majority of the patients were occupationally exposed to latex before the diagnosis, and 60% of them were health-care professionals. The distribution of health-care workers between active and placebo groups was well-balanced. Demographic and clinical features and baseline characteristics were well matched, except in the number of patients who suffered from asthma, which were predominantly allocated to the active group. Also, there was a difference in the NRL specific IgE, which was also higher in the active group.

Regarding the allergenic distribution, all the patients had a uniform pattern of latex sensitization, and recognized predominantly Hev b 5 and Hev b 6 allergens that are considered major allergens in patients occupationally exposed to latex [22]. Only 2 patients were sensitized to Hev b 1, an allergen linked to patients with spina bifida and repeated surgical procedures. The individual assessment of those cases showed that they had not a response to therapy, which could be partly attributed to the fact that Hev b 1 was not present in the composition of the extract used in the immunotherapy, according to manufacturer's information.

The study also suffered from one of the usual weakness in clinical trials in allergy comparing with other diseases, namely the lack of a single, objective, reliable and easily measurable variable to evaluate the efficacy of treatment. Therefore, in the case of immunotherapy studies, the analysis of efficacy usually relies on several clinical or analytical variables, self-questionnaires, measurements of quality of life, etc. Moreover, the particular case of latex immunotherapy has an additional difficulty comparing with clinical trials with other allergens since some of the commonly used variables (symptoms score, consumption of medication) were not useful because the patients avoid contact with the allergen and these parameters are thus not measurable. Although other authors [12] have used a clinical score to evaluate the efficacy of a latex extract, the use of such evaluation cannot be considered appropriate when no allergenic exposure is expected. However, in our study, this difficulty was overcome by the use of several *in vivo *(skin prick test, CCT, GUT) and *in vitro *tests (determination of specific IgE and BAT) to evaluate the therapeutic response to immunotherapy.

The main result of the study was the lack of significant differences in the majority of the variables studied, and in none of the different comparisons performed: comparison between groups (active *versus *placebo group after one year of treatment) and within groups after one or two years of active treatment.

The only variable with a statistically significant difference was a fall in the percentage of basophils activated in contact with NRL in BAT in the active group versus placebo. Other published studies showed a significant decreased percentage of basophils activated after 4 months of oral immunotherapy to peanut, analysing the result of one concentration (10 μg/ml) of the allergen used in the test [23]. However, the clinical significance of this finding remains unclear because no clear relationship exist with the remaining variables studied. In the future, more studies are needed that explore this finding.

The evolution of specific IgE showed an initial increase only seen when the patients received the active treatment, and a subsequent decrease in the second year of treatment in the active group, consistent with a transitory immunological effect of the immunotherapy.

Surprisingly, a previous not controlled study [11] with the same extract and in a similar number of patients (26) shows an improvement in glove-use test and rubbing test after only 10 weeks of treatment (4-day build-up phase followed by 9 weeks of maintenance). However, likewise to our trial, no change was detected by the parallel line assay for prick test. Another similar clinical [12] using the same extract in 35 patients found an improvement in symptom and medication scores in active group after12 months of treatment. The study was well-designed, but certain aspects require further clarification since no data were provided as to the occupation of the patients and the number of patients exposed to latex. Furthermore, the differences found in the study could be explained by the different exposure to NRL in both groups. In view of the low incidence of symptoms of NRL-allergic patients when they are diagnosed, we desisted from considering symptoms and medication scores as a variable. However, contrarily to our results, the authors did find a significant difference in the glove use test score between placebo and active group after 12 months of treatment.

A low frequency of adverse effects was recorded during the build-up phase of the treatment and all the patients reached the high dose. However, two patients had to drop out the study because of cutaneous symptoms that appeared during the maintenance phase. Those findings are awfully different to the reported in a very recent trial with 12 patients, in which 3 patients dropped out due to severe adverse events during induction phase [13]. Nevertheless, the explanation can rely in the fact that 2 of such patients had a history of anaphylactic events to chesnut and it is well known the association of latex sensitization with fruit allergy, including nuts.

Bearing in mind how well the treatment was tolerated, the lack of efficacy could be explained by the low dose used during the maintenance phase of the treatment. During the build-up phase, a dose of 500 mcg was reached by the fourth day, but from then on, a dose of only 40 mcg/d was administered during the rest of the treatment. It could thus be hypothesized that a greater dose should be used during the maintenance phase, especially when a long-term effect is desired. The low size of the sample, and the difference in the distribution of the patients that had suffered NRL-triggered asthma and the means of specific IgE to NRL between active and placebo group could also affect the absence of demonstrated efficacy.

## Conclusion

In conclusion, further studies are needed to evaluate the efficacy of the assayed NRL-sublingual immunotherapy. Specifically, use of a higher dose in the maintenance phase should be explored, since the maximum dose of the build-up phase has shown good tolerance. However, this present study raises some doubts as to the applicability of this immunotherapy to those patients who can avoid contact with latex.

## Competing interests

The authors declare that they have no competing interests.

## Authors' contributions

GG participated in the study design, patients' enrolment, statistical analysis and drafting of the manuscript. JA participated in the study design, statistical analysis and drafting of the manuscript. OU participated in the patients' enrolment, statistical analysis and drafting of the manuscript. MTA participated in the patients' enrolment and drafting of the manuscript. EF participated in the patients' enrolment. MLS carried out the immunoassays. DM participated in the patients' enrolment and drafting of the manuscript. All authors read and approved the final manuscript.
